# Zinc Water Prevents Autism-Like Behaviors in the BTBR Mice

**DOI:** 10.1007/s12011-022-03548-1

**Published:** 2023-01-05

**Authors:** Li Zhang, Xiaowen Xu, Liya Ma, Xinxin Wang, Meifang Jin, Lili Li, Hong Ni

**Affiliations:** Division of Brain Science, Institute of Pediatric Research, Children’s Hospital of Soochow University, Suzhou, China

**Keywords:** Autism, Zinc water, Seizure threshold, Hippocampal neurogenesis, Mossy fiber sprouting

## Abstract

This study aims to explore the effects of zinc water on autism-like behavior, convulsion threshold, and neurogenesis in ASD model animals. This study used the young BTBR ASD mouse model to explore the effect of a 6-week zinc water supplementation on ASD-like behaviors such as repetitive behavior and social communication disorder, seizure threshold, and the correlation with excitability regulation. The mice were divided into four groups of normal controls (B6) and models (BTBR) who did and did not receive zinc supplementation in water (B6, B6 + zinc, BTBR, and BTBR + zinc). For morphological changes in the hippocampus, we selected two indicators: hippocampal mossy fiber sprouting and neurogenesis. ASD-like behavior testing, seizure threshold determination, Timm staining, and neurogenesis-related assays—represented by Ki67 and DCX—were performed after 6 weeks of zinc supplementation. Our results show that zinc water can prevent autism-like behavior, reduce susceptibility to convulsions, and increase the proliferation of hippocampal progenitor cells in BTBR mice but has less effect on mossy fiber sprouting and neural progenitor cell differentiation. Zinc water reduces autism-like behavior in a partially inherited autism model mice—BTBR—which may be associated with hippocampal neural precursor cell proliferation and reversed hyperexcitability.

**Supplementary Information:**

The online version contains supplementary material available at 10.1007/s12011-022-03548-1.

## Introduction

Autism spectrum disorder (ASD), a pervasive developmental disorder, is a heterogeneous neurodevelopmental disorder whose clinical manifestations are mainly social interaction, communication disorders, and repetitive behaviors [1]. ASD prevalence is high, ranging from 0.043 to 2.68% [2]. Moreover, the prevalence of ASD is increasing annually among people of all ages and, therefore is an important public health problem worldwide [3]. Presently, the exact etiology of ASD remains unclear, although studies have shown that it is related to neuroinflammation, imbalance of excitability and inhibition, abnormal neurogenesis, and mitochondrial dysfunction [4, 5]. The relationship between trace element imbalance and autism is a new hot topic in recent ASD research [1]. Studies have found low hair zinc concentrations in 30% of children with ASD [6] and that serum zinc (Zn) levels in children with ASD are significantly lower than those in age-matched typically developing children [7]. Furthermore, prenatal zinc deficiency induces ASD-like behavior in mice [8]. Significantly, zinc deficiency affects synaptic SHANK proteins, which are thought to be the synaptic mechanism of ASD behavioral disorders [9, 10].

Zinc is the second most abundant trace metal element in the human body and essential for regulating the immune system and cell apoptosis, proliferation, and differentiation [11–14]. Presently, research on the mechanism underlying the effect of zinc supplementation on ASD is in its infancy. Increased maternal dietary zinc during pregnancy and lactation alters Shank3^-/-^-induced autism-like behaviors, possibly related to postsynaptic NMDA receptor-mediated currents and altered glutamatergic presynaptic function [15]. Zinc supplementation rescues CTTNBP2^-/-^ ASD-like behaviors, modulates the expression of synapse-associated proteins, and affects NMDAR function and signaling [16].

An imbalance between excitation and inhibition is an important pathological basis of ASD. Studies have found that GABAergic signals in the brains of patients with ASD are reduced and neural excitability is increased [17], and the combination of GABA-A and GABA-B receptor agonists can effectively attenuate ASD-like behavior in animal models [18]. Thus, regulating the imbalance between neural excitation and inhibition may effectively reverse ASD symptoms. However, reduced neural excitability has also been observed in MECP2 mutant mice, characterized by ASD-like behavior [19]. BTBR T + Itpr tf/J (BTBR) mice are an inbred strain that exhibit the core features of ASD (i.e., impaired sociability, altered vocalization, and restricted interest) and have many of the features associated with ASD and epilepsy of single-nucleotide polymorphisms [20]. Autism is often associated with epilepsy, possibly because increased brain excitability is a common pathological basis for both [21]. Methods to suppress brain excitability, such as a ketogenic diet, are effective in both epilepsy and ASD [22, 23]. Zinc-supplemented water is another means of reducing brain excitability. Previous studies by our lab have shown that a zinc-enriched water can increase the seizure threshold in animal models of developmental seizures and has an anticonvulsant effect. This is achieved by regulating the expression of molecules related to zinc ion metabolisms such as G-protein-coupled receptor 39 and zinc transporter 1 but not directly related to hippocampal mossy fiber sprouting [24]. Therefore, this study intends to further explore the effect of zinc supplementation on brain excitability and hippocampal mossy fiber sprouting in ASD BTBR mice.

Neurogenesis is involved in the occurrence and progression of ASD, and regulating adult neurogenesis may be a new approach to ASD research [25]. Neurogenesis refers to the process by which neural stem cells generate new neural cells through proliferation, migration, and differentiation and integrate into neural circuits to exert physiological functions [26]. The lower layer of granulosa cells in the hippocampal dentate gyrus (DG) is one of the two most important regions for adult neurogenesis in mammals [27]. Notably, the hippocampus is an important brain region involved in memory, learning, emotion, and cognition [28]. Abnormal neurogenesis induced by valproic acid (VPA) exposure during pregnancy has been observed in a variety of mice ASD models, including BTBRand SHANK3 [29–31]. Studies aimed at modulating abnormal hippocampal neurogenesis in ASD mouse models have also shown better changes [32]. Furthermore, aberrant adult hippocampal neurogenesis alters the excitability of mature dentate granule neurons [33]. However, currently, there have been no reports on the intervention effect of zinc supplementation on neurogenesis in ASD mice.

The present study shows that 6 weeks of zinc water supplementation may alleviate autism-like behavior in BTBR mice by reducing brain excitability and promoting the proliferation of hippocampal neural progenitor cells, which provides a morphological basis for the effect of zinc supplementation on reducing excitability in ASD mice.

## Materials and Methods

### Animals

BTBR mice were purchased from Jackson Laboratory in the USA (#002282), and B6 (C57BL/6, referred to as B6) mice were purchased from the Experimental Animal Center of Soochow University. All procedures were approved by the Regulations for the Administration of Laboratory Animals (NO.SUDA20220617A01). Newborn mice were weaned on the 21st day. Males (excluding females) were then fed in separate cages until day 63, with no more than five mice per cage.

### Zinc Water Management

All experimental animals were randomly assigned to the normal control (B6, *n*=32), zinc water control (B6 + zinc, *n*=32), model (BTBR, *n*=34), or zinc water model (BTBR + zinc, *n*=34) groups after weaning. The specific use of animals is detailed in the supplementary material. The B6 and BTBR groups were administered ddH_2_O, whereas the B6 + Zinc and BTBR + zinc groups were given ddH2O containing 60 ppm zinc provided by ZnSO4·7H2O. Furthermore, the mice were fed with the irradiated feeds from Suzhou Shuangshi Lab Animal Feed Technology Co(#D10012G). The content of some metallic elements is shown in Supplementary Table 1. Behavioral tests and other experiments were performed after 6 weeks of Zn-water administration [34, 35].

### Testing

At 63 days old, the mice were subjected to 5 days of behavioral testing, with one test per day. The order of testing was randomized for each mouse, and the experimental setup is shown in Fig. 2A. The testing time was from 9:00 am to 6:00 pm. All tests were recorded by video equipment (Huawei, China) and measured manually by analysts using stopwatches and counters. Neither the testers nor analysts were aware of the composition of the experimental groups. For behavioral tests, 12 mice were randomly selected from each group using the numerical table method.

### Body Weight

Body weight was measured every 7 days from 21 to 63 days.

### Open Field Test

The open field test lasted 5 min and was performed as previously described [16] to evaluate anxiety and motor activity in mice. A 40 cm × 40 cm × 30 cm acrylic glass box was used, equally divided into 16 squares. The central area was defined as a square area of 20 cm × 20 cm equidistant from the wall of the box, a total of 4 squares, and the remaining 12 squares were defined as the surrounding area. The movement of the experimental mice was measured by the number of times they crossed the grid, defined as from entering with all limbs to leaving with all limbs. The time to enter the central grid during the open field activity was used as a measure of anxiety-like behavior, with smaller values indicating greater anxiety [36]. The number of gridlines across in the open field test was used to describe distance of movement, with higher values indicating increased athletic ability. The number of urination, defecation, and standing events were also recorded.

### Self-Grooming

Grooming experiments were performed as described previously [37] to evaluate repetitive stereotyped behaviors in mice. An empty transparent glass cylinder of 20 cm in diameter and 20 cm in height was used. After the mice were acclimated in the glass cylinder for 10 min, any self-grooming behavior (grooming any part of their body with their mouth or forelimbs) during the next 10 min was recorded.

### Marble-Burying

The marble-burying test was performed as described in the literature [38] to evaluate the repetitive behavior of mice. Fifteen black glass marbles with a diameter of 15 mm were gently placed in a symmetrical and equidistant manner in an experimental mouse cage lined with 3-cm-thick granular bedding. The number of marbles buried by the mice within 30 min was recorded, and marble burial was defined as approximately 75% of the volume of a marble buried in the bedding.

### Y-maze

As described in the literature [39], the Y-maze test was used to assess repetitive behavior and activity in mice. The maze had a length, width, and height of 30 cm, 5 cm, and 15 cm, respectively, for each arm. The total number of entries was the number of entries into maze arms. The actual alternations were the total number of consecutive entries into all three arms, and the alternation ratio was calculated as total alternations/(total number of entries-2).

### Three-Chamber Experiment

As described in the literature [16], a three-chamber experiment was used to evaluate the social interaction of the mice. The equipment used for this test was a rectangular acrylic glass apparatus of length × width × height 60 cm × 20 cm × 20 cm, divided into three equal-volume chambers by two movable transparent glass plates. The effective sniffing range was defined as 2 cm around the metal cage. In the social experiment, a plastic toy, similar in size to the mouse to be tested, was placed in an acrylic glass cage, which was labeled Ob. A mouse S1 of the same age, sex, weight, and strain as the mouse to be tested was placed in the cage on the other side. The time spent sniffing Ob and S1 within 10 min was recorded as TOb and TS1, and the social preference index is defined as (TS1-TOb)/(TS1 + TOb). After the mouse to be tested had a 5-min rest, Ob was replaced by another mouse of the same age, sex, and weight as the mouse to be tested, recorded as S2. The time spent by the mouse to be tested sniffing S1 and S2 during 10 min was recorded as TS1 and TS2, and the novelty preference index was defined as (TS2 − TS1)/(TS1 + TS2).

### Seizure Threshold

This experiment was conducted as previously described [40]. Briefly, mice were timed immediately after intraperitoneal injection of penicillin solution (5.1×10*6U/kg, Sangon Biotech, China), convulsions were observed, and the time from the start of injection to grade IV and above seizures was defined. For seizure threshold, a total of 90 min was observed, and the seizure threshold was 90 min when no convulsions occurred. Convulsive seizure grades (Supplementary Table 3) were determined according to the modified Racine scale [41]. This experiment included 48 male mice (12 per group) that were used only for the seizure threshold measurements.

### Measurement of Zinc Concentration in Serum

The zinc concentration in serum was operated according to the kit instructions (#BC2815, Solarbio), and the specific procedure is added in the supplementary material.

### Timm’s Staining

In each group, 4 mice were randomly selected and anesthetized with 1% sodium pentobarbital (75 mg/kg) for cardiac perfusion. The perfusion liquid consisted of 10 ml PBS, 10 ml 0.4% sodium disulfide solution, 10 ml 4% paraformaldehyde solution, and 10 ml 0.4% sodium disulfide solution. The brain tissue was placed in 4% paraformaldehyde solution overnight; dehydrated in 10%, 20%, and 30% sucrose solutions; embedded with OCT; and then stored at −80°C overnight. The blocks were quickly transferred to a cryostat for slicing (30 μm thickness) and stored at −20°C. Frozen sections were returned to room temperature in the dark and then placed in a dark–dark staining solution tank containing 60 ml of Timm’s staining solution for 1 h at room temperature. Sections were rinsed with double-distilled water for 30 min, dried, dehydrated with graded alcohol, and sealed with neutral gum. Three nonconsecutive sections of each brain tissue were observed with a microscope (NikonE100, Japan), and the distance between each section was 200 μm. The observation sites were the DG and CA3 areas, and staining was evaluated according to the Gavazos mossy fiber sprouting (MFS) standard (Supplementary Table 4) [42].

### Immunofluorescence Staining

The brains of mice (*n*=4 in B6 and B6 + zinc groups; *n*=6 in BTBR and BTBR + zinc groups) were used for immunofluorescence staining. As described previously [43], fresh tissue samples obtained after anesthesia overdose (1% sodium pentobarbital, 225 mg/kg) were placed in 4% paraformaldehyde solution overnight at room temperature, dehydrated in graded sucrose solutions, and embedded in paraffin. Paraffin sections of 4 μm thickness were sliced and dewaxed and subjected to gradient dehydration. Sections were used for antigen retrieval with EDTA antigen retrieval buffer (PH8.0), incubated with 5% BSA for 30 min, and then incubated with primary antibody (DCX 1:100,#GB11317, Anti-Rabbit, Servicebio; Ki67 1:500,#GB111499, Anti-Rabbit, Servicebio) at 4°C overnight. After washing, sections were incubated with secondary antibody (1:400, Servicebio) for 60 min at room temperature, dried, and mounted with anti-quenching DAPI (Boster, Wuhan). Three nonconsecutive slices of each brain were observed, with a distance of 100 μm between each slice, and a photomicrograph was randomly selected for ImageJ quantification.

### Statistical Methods

GraphPad Prism 7.0 (GraphPad Software, San Diego, CA, USE) was used for statistical analysis of experimental results, and graphs were drawn. Data are presented as mean ± standard deviation (mean ± SD). Paired *t* tests were used for the interaction time with Ob-S1 and S1-S2 (Fig. 2) except for the BTBR group in Ob-S1 where the Wilcoxon signed-rank test was used. Weight and weight growth rates were analyzed using a three-way ANOVA. Seizure thresholds were tested using the Kolmogorov–Smirnov test. Convulsive grade and Timm staining score were tested using the Kruskal–Wallis test. The Scheirer–Ray–Hare test was used for seizure thresholds. Two-way ANOVAs were used for the rest of the data except those listed above. The Spearman test was used for the correlation test between the two variables.

## Results

### Zinc Concentration in Serum Increased After Zinc Water Administration

**Fig. 1:**
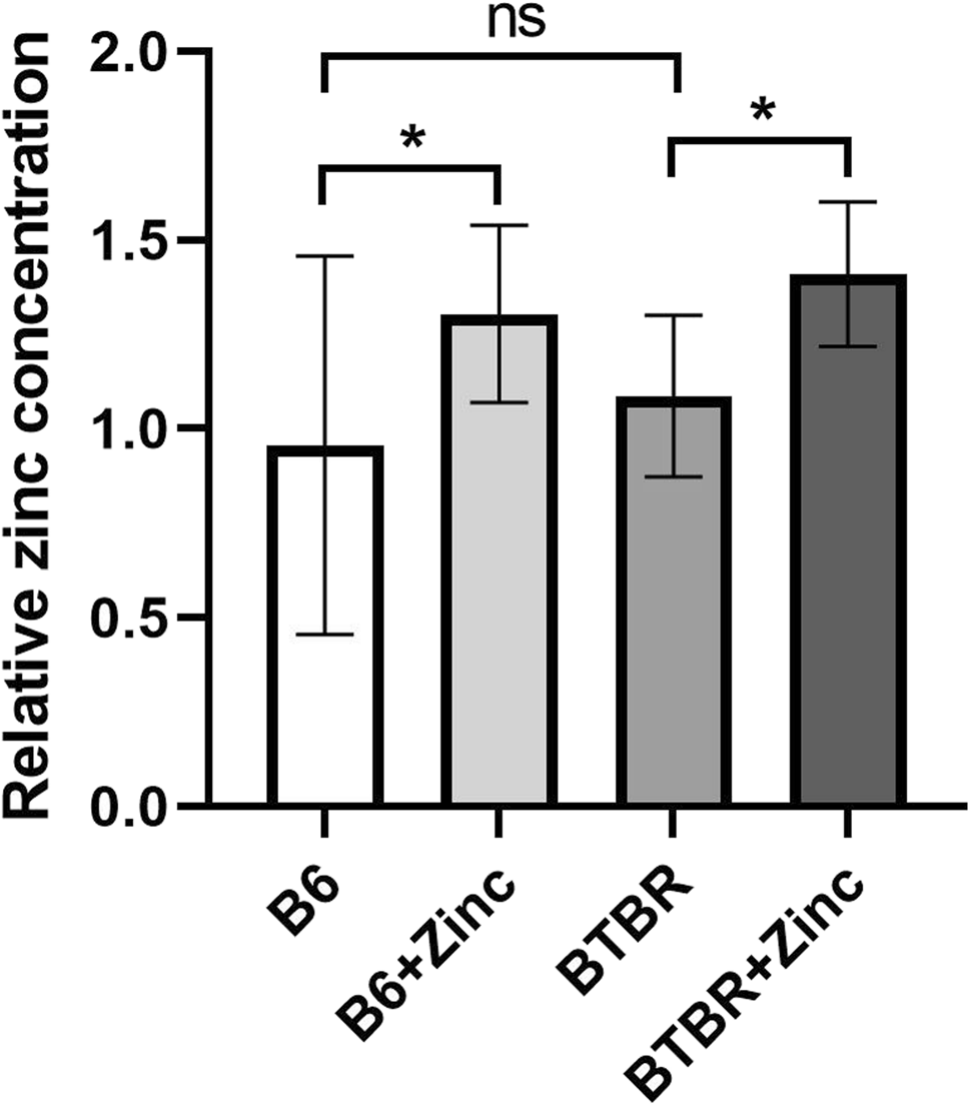
Serum zinc concentration increased after zinc water administration. Serum zinc concentration relative to group B6 (*n*=12/group). Analyzed using two-way ANOVA with Holm–Šídák multiple comparisons test. * *p* < 0.05

Serum zinc concentration after zinc water administration was mainly influenced by zinc water (Fig. 1) (*F*(1, 40) = 12.72, *p* < 0.01), and the effect of genotype and genotype × zinc water was not significant. Serum zinc concentrations increased after zinc water administration in B6 and BTBR mice (*p*<0.05).

### Zinc Supplementation Has Little Effect on Body Weight

**Fig. 2:**
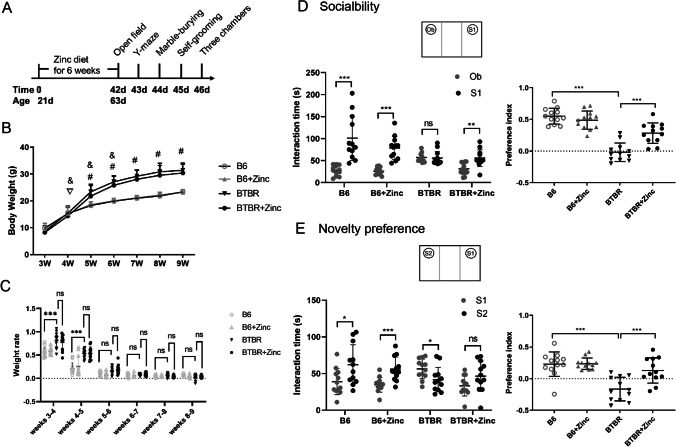
Effect of zinc supplementation on body weight, weight rate, and the three-chamber experiment. **A** Experimental procedure; **B** body weight changes over the 6 weeks of supplementation analyzed using three-way ANOVA; and **C** weight rate changes over the 6 weeks of supplementation analyzed using three-way ANOVA; **D** social and **E** novelty part of the three-chamber experiment (*n*=12/ group). Paired *t* test was used for the interaction times except for Ob-S1 in the BTBR group, while two-way ANOVA with Holm–Šídák multiple comparisons test was used for the preference indices.▽, 3-week B6 vs 4-week B6, *p*< 0.05; #, BTBR vs B6, *p*< 0.05; &, (*x*+1) weeks BTBR vs *x* weeks, *x*=5, 6, 7, 8, *p*< 0.05; ns, no statistical significance, *p*> 0.05, * *p*< 0.05, ** *p*< 0.001, *** *p*< 0.001

We observed the effect of the zinc supplementation on body weight in B6 and BTBR mice weekly for 6 weeks (Fig. 2B, C). The three-way ANOVA showed that time, genotype, zinc water, and genotype × time significantly affected body weight (Table 1). Time, genotype, and genotype × time had a significant effect on growth rate (Table 2). Post hoc analysis showed that for both the B6 and BTBR strains, body weight was significantly heavier at week 4 for the B6 than at week 3 (*p*<0.05), with no statistical difference for the other weeks compared to that of the previous week (*p* were all greater than 0.05). BTBR had significantly heavier body weight at weeks 4–6 compared to that in the previous week (*p*<0.05), with no difference in body weight at weeks 7–9 compared to that in the previous week (*p*>0.05). There was no difference in body weight between the BTBR strain and the B6 strain compared to the B6 strain at weeks 3 and 4 (*p*>0.05), and from week 5 until the end of the observation at week 9, the BTBR strain was significantly heavier than the B6 strain. BTBR had a significantly faster growth rate than B6 at weeks 3–4 and 4–5 (*p*<0.05). However, there was no statistical difference in the effect of zinc water on body weight and growth rate compared to the respective controls for both B6 and BTBR (*p*>0.05).

**Table 1:** Three-way analysis of body weight

	Factor	DFn	DFd	F	*p*
Three-way	Genotype	1	308	321.2	*p*<0.0001*
ANOVA	Zinc water	1	308	4.385	*p*=0.0371*
	Time	6	308	372.6	*p*<0.0001*
	Genotype × zinc water	1	308	5.067	*p*=0.0251*
	Genotype × time	6	308	33.68	*p*<0.0001*
	Zinc water × time	6	308	0.01993	*p*>0.9999
	Genotype × zinc water × time	6	308	0.1950	*p*=0.9781

* *p*<0.05

**Table 2:** Three-way analysis of weight rate

	Factor	DFn	DFd	F	*p*
Three-way	Genotype	1	264	92.94	*p*<0.0001*
ANOVA	Zinc water	1	264	0.3485	*p*=0.5555
	Time	5	264	380.4	*p*<0.0001*
	Genotype × zinc water	1	264	0.03851	*p*=0.8446
	Genotype × time	5	264	23.43	*p*<0.0001*
	Zinc water × time	5	264	0.1800	*p*=0.9700
	Genotype × zinc water × time	5	264	0.7854	*p*=0.5609

* *p*<0.05

### Zinc Supplementation Increases the Sociability of BTBR Mice in a Three-Chamber Socialization Experiment

To determine whether zinc supplementation could alleviate social deficits in BTBR mice, three-chamber socialization experiments were used. In the social part of the three-chamber experiment (Fig. 2D), the effects of genotype (Fig. 2D) (*F*(1, 44) = 6.945, *p* < 0.001) and zinc water (Fig. 2D) (*F*(1, 44) = 78.07, *p* < 0.001) on the socialization ability of mice were significant. Furthermore, there was an interaction between genotype × zinc water (Fig. 2D) (*F*(1, 44) = 19.41, *p* < 0.05). The Holm–Šídák test showed that the social preference index was significantly lower for BTBR than that for B6 (*p* < 0.05). Zinc water had little effect on the social preference index of B6 but significantly increased the social preference index of BTBR. Consistently, B6 preferred murine S1 to objects (Ob) (*p* < 0.05), while BTBR showed little difference in the sniffing time between Ob and S1 (*p* > 0.05). However, zinc water caused BTBR to spend more time interacting with S1 than with Ob.

During the novelty preference phase of the three-chamber experiment, genotype (Fig. 2E) (*F*(1, 44) = 6.935, *p* < 0.05), zinc water (Fig. 2E) (*F*(1, 44) = 34.02, *p* < 0.001), and genotype × zinc water (Fig. 2E) (*F*(1, 44) = 15.87, *p* < 0.001) were the main factors to influence the social novelty index. The Holm–Šídák test showed that the social novelty index was significantly reduced in BTBR compared to that in B6. Zinc water had little effect on the social novelty index of B6, although it could significantly increase the social novelty index of BTBR. Concordantly, B6 mice spent more time interacting with the unfamiliar mouse S2 than with the familiar mouse S1 (Fig. 2E) (*p* < 0.05). BTBR, on the other hand, tended to interact more with S1 than with S2, while zinc water altered this tendency. In conclusion, zinc water treatment increased the sociality of BTBR.

### Zinc Supplementation Reduces Repetitive Behavior in BTBR Mice in the Marble-Burying Test and Self-Grooming But Has Less Effect on Repetitive Behavior in the Y-Maze

**Fig. 3:**
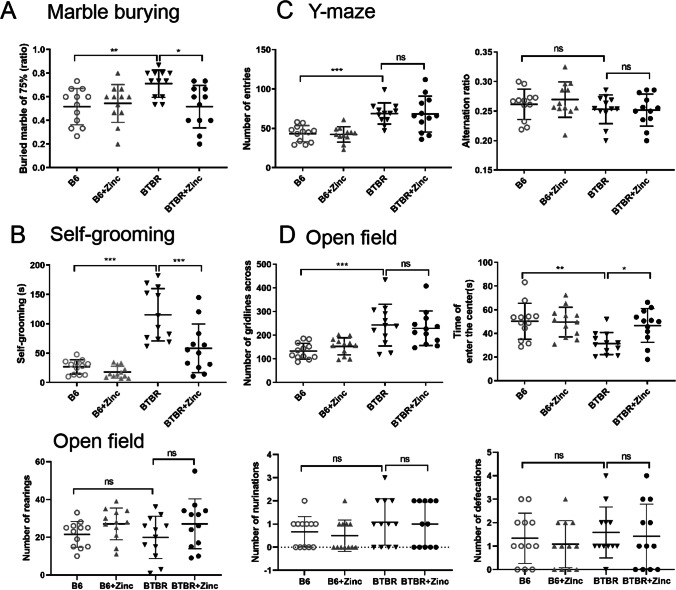
Zinc supplementation prevents some repetitive and anxiety-like behavior in BTBR mice. **A** Marble-burying test; **B** grooming test; **C** Y-maze; **D** open field test (*n*=12/group). Analyzed using two-way ANOVA with Holm–Šídák multiple comparisons test. * *p*< 0.05, ** *p*< 0.001, *** *p*< 0.001

In the marble-burying test, the main effects of genotype (Fig. 3A) (*F*(1, 55) = 4.964, *p* < 0.05) and zinc water (Fig. 3A) (*F*(1, 55) = 7.338, *p* < 0.01) were significant. BTBR had a higher rate of marble burial than did B6 (Fig. 3A) (*p* < 0.01), while zinc water could reduce this rate in BTBR (Fig. 3A) (*p* < 0.05).

In the self-grooming test, two-way ANOVA revealed genotype (Fig. 3B) (*F*(1, 45) = 53.36, *p* < 0.001), zinc water (Fig. 3B) (*F*(1, 45) = 14.06, *p* < 0.001), and an interaction between genotype × zinc water (Fig. 3B) (*F*(1, 45) = 7.483, *p* < 0.01) were statistically significant. The Holm–Šídák test showed that self-grooming time was significantly increased in BTBR mice compared with B6 mice (Fig. 3B) (*p* < 0.001), whereas zinc supplementation reduced self-grooming time in BTBR mice (Fig. 3B) (*p* < 0.001).

However, in the alternation ratio in the Y-maze, two-way ANOVA showed no effects from zinc water (Fig. 3C) (*F*(1, 44) = 0.1696, *p*=0.6824), genotype (Fig. 3C) (*F*(1, 44) = 2.889), *p*=0.0962), or interaction between these two factors (Fig. 3C) (*F*(1, 44) = 0.3733, *p*=0.5444). Contrastingly, in the total alternation in the Y-maze, a genotype effect was observed (Fig. 3C) (*F*(1,44) = 35.55, *p*<0.0001). There was no statistically significant difference for zinc water (Fig. 3C) (*F*(1,44) = 0.03723, *p*=0.8479) or interaction between these two factors (Fig. 3C) (*F*(1,44) = 0.001489, *p*=0.9694). Thus, compared to B6, BTBR mice had increased the number of entries in the Y-maze (Fig. 3C) (*p* < 0.05), but zinc supplementation did not decrease it (Fig. 3C) (*p* >0.05). Taken together, these behavioral data indicated that zinc supplementation significantly attenuated some of the abnormal repetitive behaviors in BTBR.

### Zinc Supplementation Reduced Anxiety-Like Behavior in BTBR Mice But Not Active Movement in the Open Field Test

Anxiety was determined by time of entry into the central zone. The effect of genotype (Fig. 3D) (*F*(1, 45) = 8.391, *p* < 0.01), zinc water (Fig. 3D) (*F*(1, 45) = 4.071, *p* < 0.05), and interactions of genotype × zinc water (Fig. 3D) (*F*(1, 45) = 4.494, *p* < 0.05) on the time of entry into the center were significant. Compared with B6, BTBR mice were more anxious (Fig. 3D) (*p* < 0.05), whereas zinc supplementation reduced anxiety in BTBR mice (Fig. 3D) (*p* < 0.05).

Movement distance was measured by the grid bars. Two-way ANOVA showed the effect of genotype on movement distance was statistically significant (Fig. 3D) (*F*(1, 44) = 24.98, *p*<0.001), whereas the zinc water (Fig. 3D) (*F*(1, 44) = 0.001864, *p*=0.9658) and genotype × zinc water (Fig. 3D) (*F*(1, 44) = 0.6739, *p*=0.4161) were not. Compared to B6 mice, BTBR mice showed increased movement distance (Fig. 3D) (*p* < 0.001), whereas zinc supplementation did not attenuate this activity in BTBR mice. The number of mice standing, urinating, and defecating was not statistically significant. In conclusion, zinc supplementation alleviates the anxiety-like behavior in BTBR mice but did not attenuate active movement in the open field test.

### Zinc Supplementation Has Little Effect on the Differentiation of Hippocampal Neural Progenitor Cells in BTBR Mice

**Fig. 4:**
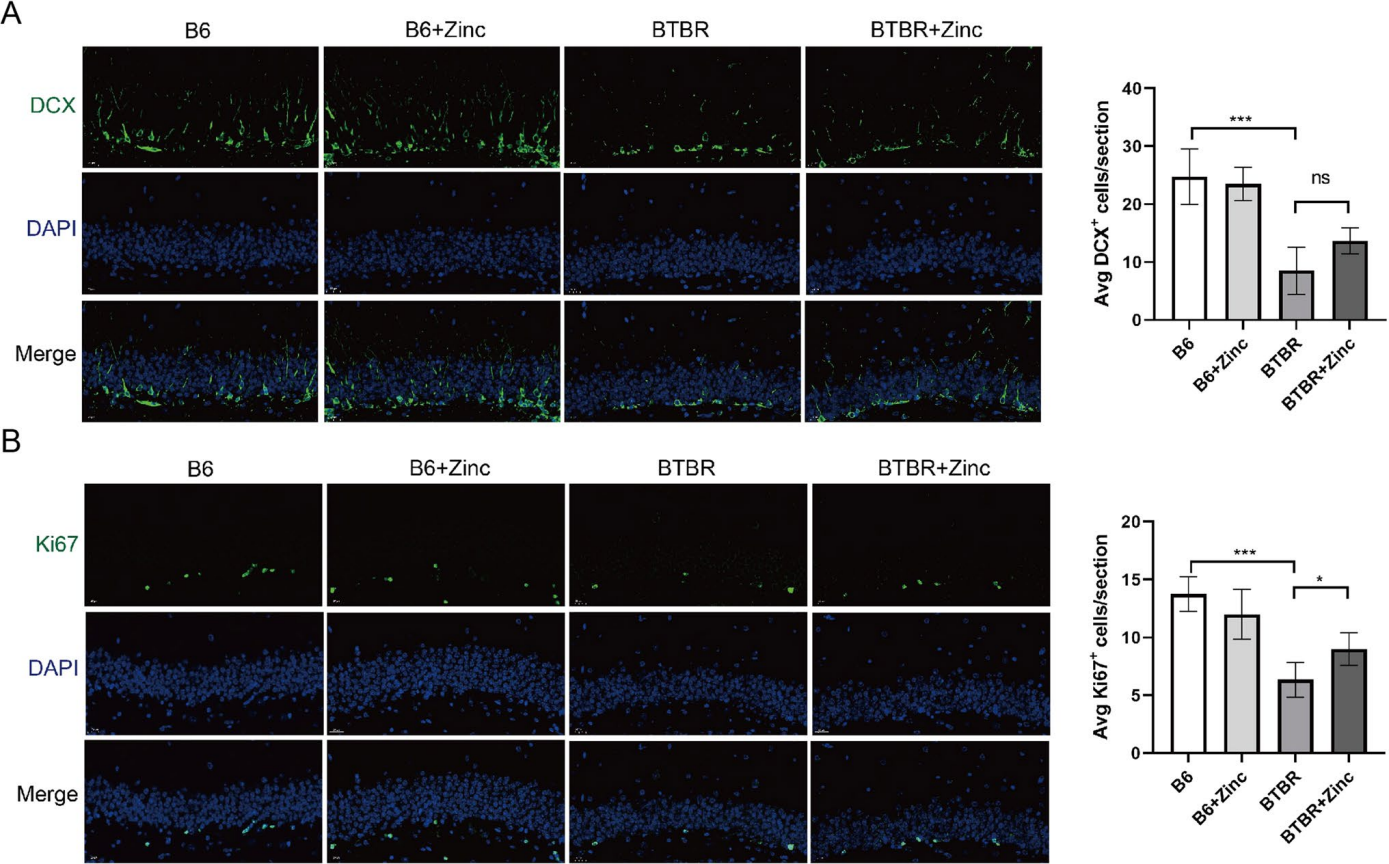
Effects of zinc supplementation on hippocampal neurogenesis. **A** Immunofluorescence and quantitative analysis of DCX. **B** Immunofluorescence and quantitative analysis of Ki67. B6 group *n*=4; *n*=6 B6 + zinc, BTBR, and BTBR + zinc group. Statistically analyzed using two-way ANOVA with Holm–Šídák multiple comparisons test. * *p*< 0.05, *** *p*< 0.001

DCX was used to assess neural progenitor differentiation in the DG of the BTBR mice. Two-way ANOVA revealed a genotype effect (Fig. 4A) (*F*(1,16) = 64.48, *p*<0.001) on DCX+ cell numbers. However, zinc supplementation (Fig. 4A) (*F*(1, 16) = 1.454, *p*=0.2454) and genotype ×zinc water (Fig. 4A) (*F*(1, 16) = 3.902, *p*=0.0657) had no significant effect on DCX+ cell numbers. The number of DCX cells in the DG of BTBR was significantly reduced when compared to that of B6. However, these data showed that zinc supplementation had little effect on neuronal differentiation in the hippocampus of BTBR mice.

### Zinc Water Treatment Increases Hippocampal Neural Progenitor Cell Proliferation in BTBR Mice

Ki67 was used to assess neural progenitor proliferation in the DG of the BTBR mice. Two-way ANOVA revealed that genotype (Fig. 4B) (*F*(1, 16) = 49.50, *p* <0.001) and genotype ×zinc water (Fig. 4B) (*F*(1,16) = 8.900, *p* <0.01) were significantly different in Ki67+ cells. However, the effect of zinc supplementation (Fig. 4B) (*F*(1, 16) = 0.3834, *p*=0.5445) on the number of Ki67+ cells was not statistically significant. Statistical analysis showed that Ki67+ cells in BTBR (Fig. 4B) (*p* < 0.001) were significantly reduced in the DG compared to B6. In other words, proliferation of hippocampal neurons was impaired in BTBR. Zinc supplementation increased the number of Ki67+ cells in BTBR mice (Fig. 4B) (*p* < 0.05). These data indicate that zinc supplementation significantly restored neuronal proliferation in the hippocampi of BTBR.

### Zinc Supplementation Has Little Effect on Timm Staining Results

**Fig. 5:**
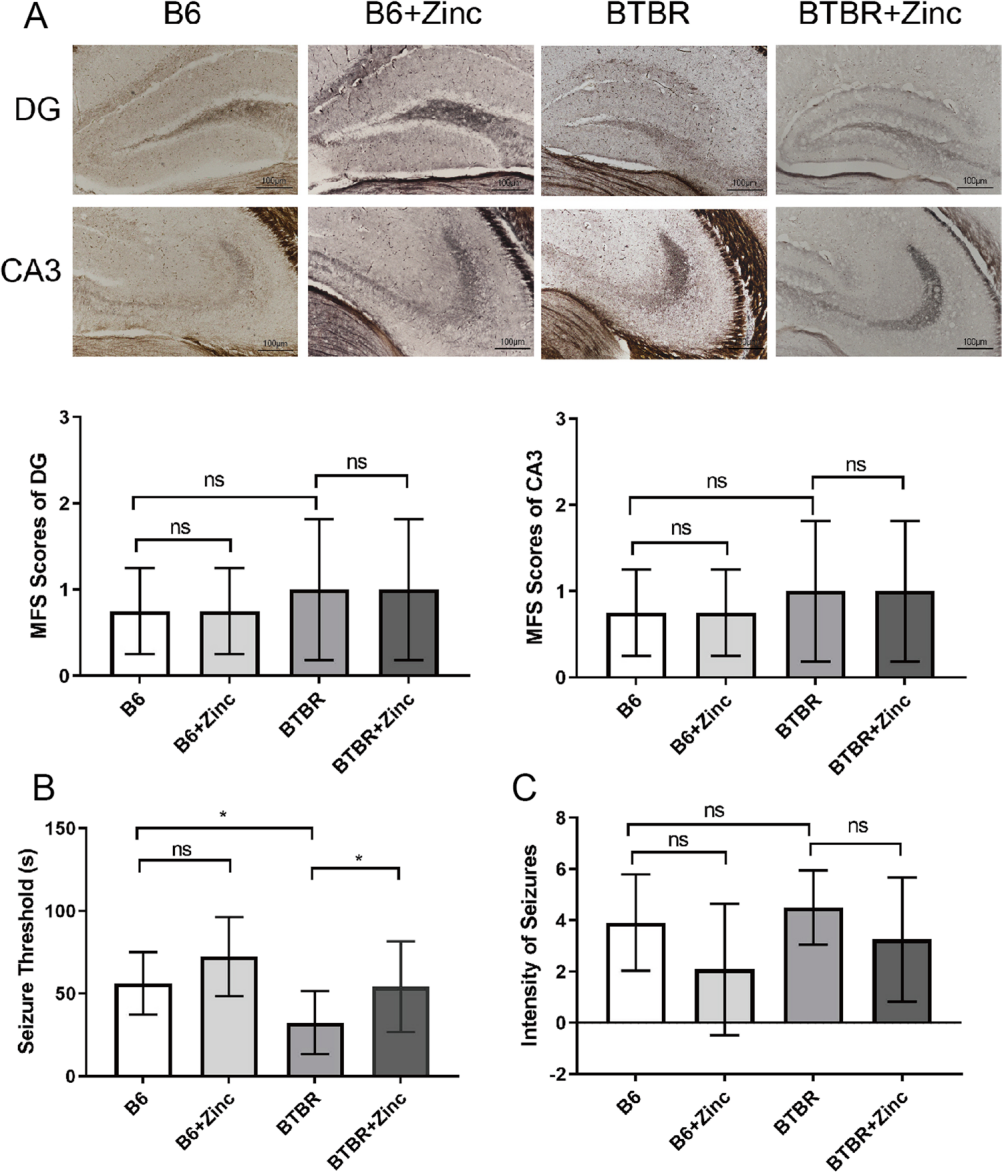
Zinc supplementation can increase the convulsion threshold of BTBR mice, but the effect on Timm staining was not statistically significant. **A** Timm staining (×200; *n*=4/group) and Mossy fiber sprouting (MFS) score map. The MFS was used to evaluate Timm staining; **B** convulsion threshold (*n*=12/ group); **C** convulsion grade statistical map (*n*=12/ group). **A**–**C** used the Scheirer–Ray–Haretest. ns, no statistical significance, *p*> 0.05, *p< 0.05

In the DG and CA3 areas, there was little difference between the groups in terms of mossy fiber distribution and germination indicators (Fig. 5A) (*p* all greater than 0.05).

### Zinc Water Prolongs Convulsion Threshold

The convulsive threshold was used to determine seizure threshold. The Scheirer–Ray–Hare test showed genotype (Fig. 5B) (*H* = 10.4059, *p*=0.0013) and zinc water (Fig. 5B) (*H* = 5.4197, *p*=0.011) had major effects on the seizure threshold. The convulsion threshold of BTBR mice was lower than that of B6 (*p*< 0.05), and zinc supplementation significantly restored the convulsion threshold of BTBR (Fig. 5B) (*p*< 0.05). There was no statistically significant difference between groups in the seizure grade (Fig. 5) (*p* all greater than 0.05).

### Alterations in Convulsive Thresholds and Ki67 Were Associated with the Remission of Some Autistic-Like Behaviors

**Fig. 6:**
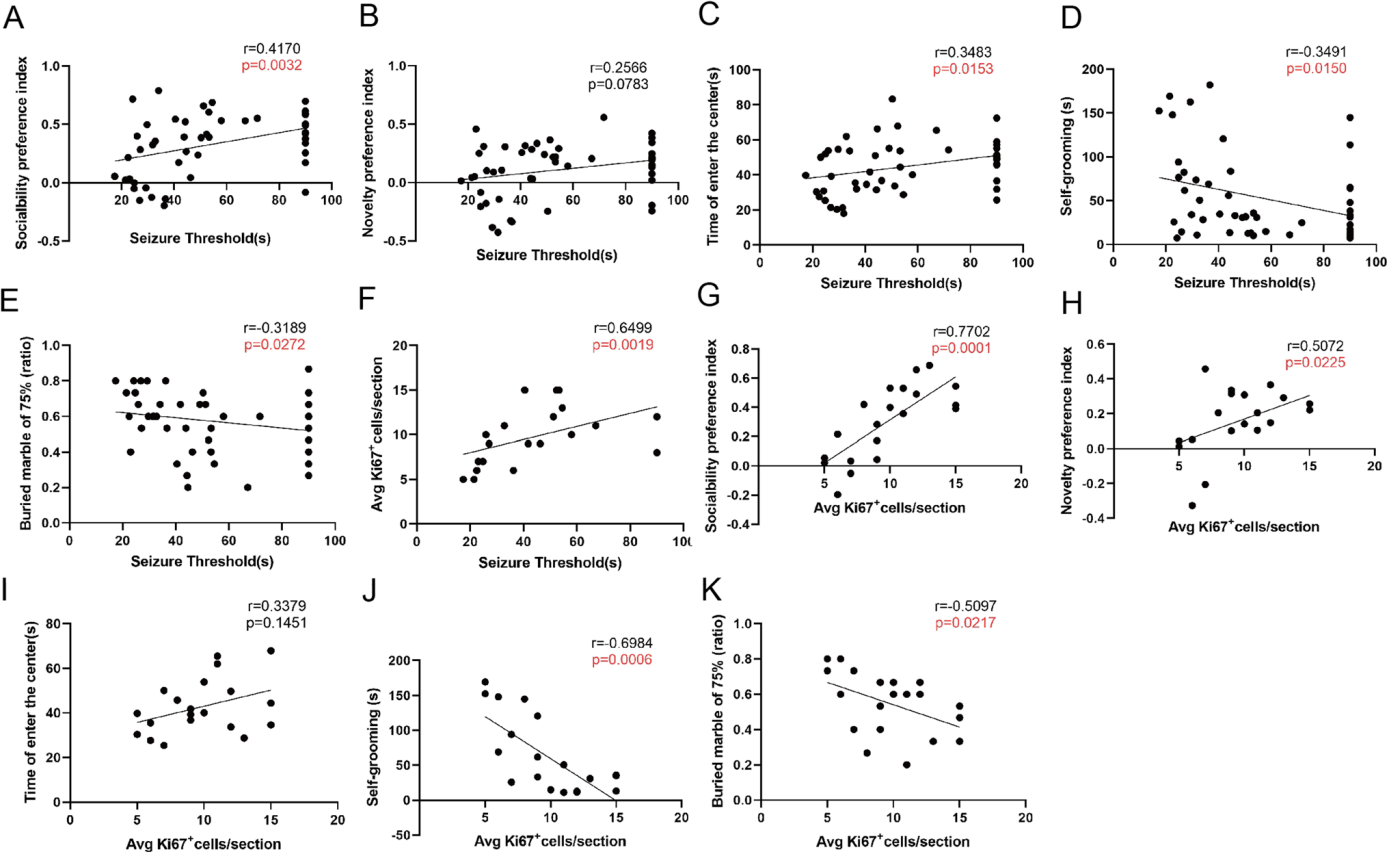
Alterations in convulsive thresholds and Ki67^+^ cells were associated with the remission of some autistic-like behaviors. **A**–**K** used the Spearman test. **A** Positive correlation between seizure thresholds and social preference index. **B** No correlation between seizure thresholds and novelty preference index. **C** Positive correlation between seizure thresholds and time of enter center. **D** Negative correlation between seizure thresholds and self-grooming. **E** Negative correlation between seizure thresholds and marble burial of 75%. **F** Positive correlation between seizure thresholds and ki67Ki67^+^ cells. **G** Positive correlation between Ki67^+^ cells and social preference index. **H** Positive correlation between ki67 and novelty preference index. **I** No correlation between ki67 and time of enter center. **J** Negative correlation between ki67 and self-grooming. **K** Negative correlation between ki67Ki67^+^ cells and marble burial of 75%

To further investigate the relationship between neural progenitor cell proliferation in hippocampal DG regions, convulsive threshold, and altered autistic-like behavior, correlation analyses were performed (Fig. 6). Convulsive thresholds were positively correlated with social preference in autistic-like behaviors and time to enter the central area in the open field and negatively correlated with marble burial and self-grooming (Fig. 6A, C–E). However, they were not correlated with social novelty (Fig. 6B). Ki67 was positively correlated with social preference in autistic-like behavior and negatively correlated with marble burial and self-grooming (Fig. 6G, J, K). However, it was not correlated with social novelty or with time to enter the central area in the open field (Fig. 6H, I). In addition, convulsion thresholds were positively correlated with Ki67 (Fig. 6F).

## Discussion

This study investigated the effects of zinc supplementation on autism-like behavior, seizure threshold, mossy fiber sprouting, and neurogenesis in a BTBR autism mouse model. This study found that 6 weeks of zinc supplementation which was increased zinc concentration of blood prevented autism-like behaviors, such as social impairment, repetitive behaviors, and anxiety-like behavior in BTBR mice, consistent with the excessive grooming behavior of adult Shank3^-/-^ mice offspring induced by maternal zinc supplementation [15]. More importantly, we found that zinc water decreased the seizure threshold while enhancing the proliferation of hippocampal neural cells, but no statistical difference in the abnormal sprouting of hippocampal mossy fibers and differentiation of hippocampal neural progenitor cells were found.

The balance of neural excitation and inhibition (E/I imbalance) is considered one of the neural mechanisms underlying ASD [44], with evidence of decreased GABA-A receptor density and decreased inhibition in the hippocampus of patients with ASD [45]. Another study showed that serum glutamate levels and neural excitation increased in patients with ASD [46]. Epilepsy frequently co-occurs with ASD, with 21.5% (1/4.65) of patients with ASD being diagnosed with epilepsy [47, 48]. The prevalence of epileptiform activity (EA) on electroencephalography (EEG) in patients with ASD ranges from 23.6 to 60.8% [49]. Deficiency of ASD risk factor ASH1L causes seizures in experimental mice [50]. Patch-clamp results of Fmr1-knockout hippocampal pyramidal neurons modeled by fragile X mental retardation protein (FMRP) showed decreased action potential (AP) thresholds and reduced post-mediator hyperpolarization accompanied by hyperexcitability of hippocampal neurons [51]. We examined the susceptibility to epilepsy in BTBR mice and found a higher susceptibility to convulsions than B6 mice, while zinc supplementation could reduce the convulsion susceptibility in BTBR mice. Consistent with our study, susceptibility to convulsions was increased in VPA-induced autism model mice compared to B6 mice [52]. A previous study by our group showed that zinc supplementation can reduce the seizure susceptibility of epileptic mice [24]. Conversely, zinc deficiency increases susceptibility to kainic acid-induced epilepsy, which may be associated with increased glutamate release and decreased GABA concentration [53, 54]. Contrastingly, intrahippocampal zinc injection induces seizures [55], whereas zinc chelation reduces seizures [56]. The difference may be related to alterations in the intestinal microbiota and brain–gut axis, as zinc has been shown to be a key regulator of gastrointestinal development and microbiota composition [57].

Mossy fiber axonal sprouting, that is, the axonal branching of granulosa cells abnormally or reversely projecting to the inner third of the molecular layer which builds excitatory synaptic connections with granulosa cells, forms repeated excitatory circuits, and enhances neural network excitability is considered to be one of the mechanisms of epilepsy [58]. However, it has also been suggested that mossy fiber sprouting may not cause seizures [59]. This may be related to the modulation of mental behavior, such as mossy fiber sprouting—also observed in bipolar disorder [60]. In this study, there was no significant difference in sprouting scores among the groups in Timm staining, and no mossy fiber sprouting was observed. This suggests a weak relationship between mossy fiber sprouting and susceptibility to seizures in ASD. Additionally, the low sensitivity of Timm staining cannot be excluded; therefore, slight changes may not be detected. Importantly, altered microbiota, all-inflammatory responses, and altered gut–brain signaling are also influential factors that cannot be ignored.

Several studies have shown that abnormal neurogenesis is involved in ASD development and is an important physiological and pathological change in ASD [61]. Autopsies have shown that some patients with ASD have defects in hippocampal neurogenesis and abnormalities in neuronal migration and maturation [25]. Aberrant hippocampal neurogenesis has also been observed in several ASD mouse models. In the VPA model, hippocampal neurogenesis was strongly activated early, followed by a sharp decrease in later stages [30]. Adult hippocampal neurogenesis is impaired in Ube3a transgenic mice [62]. The ventral hippocampus of Shank3 ex21 mice and the adult hippocampus of FMR1 transgenic mice were also dramatically reduced [63]. This suggests that reduced neurogenesis is associated with ASD-like behavior. Furthermore, disturbances in adult hippocampal neurogenesis lead not only to abnormal social skills but also to increased repetitive behaviors and anxiety [64]. Consistent with previous studies [31, 65], BTBR mice showed restricted neurogenesis in the adult hippocampus and a significant reduction in the number of Ki67+ cells—a marker of cell proliferation, and DCX+ cells, a marker of cell differentiation—compared with B6. Zinc water (60 ppm for 6 weeks) increased the number of Ki67-positive cells in BTBR mice. Consistent with our study, Cope et al. [29] found that in a traumatic brain injury (TBI) model, zinc supplementation (180 ppm) for 4 weeks showed significantly more EdU+ cells than that of the control group. Contrastingly, adult male mice fed a zinc-restricted diet (1 mg/kg for 3 weeks) showed a 50% reduction in Ki67+ cells in the subgranular zone (SGZ) and granulosa cell layer of the dentate gyrus, and the number of TUNEL+ cells in the SGZ also increased significantly [66]. However, in a mouse model of obesity, 4 weeks of low-dose Zn supplementation (15 ppm) reversed proliferation, neuronal differentiation, and BDNF levels. However, 4 weeks of high-dose (60 ppm) supplementation exacerbated the reduction in hippocampal neurogenesis and synaptic plasticity [67]. In the mouse obesity model, researchers observed inhibition of neurogenesis in mice supplemented with 60 ppm zinc water continuously for 4 weeks during adulthood. However, in the present study, zinc water was supplemented 21 days after birth, which may be the main reason for this difference. In addition, the differential effects of zinc supplementation on hippocampal neurogenesis may be related to differences in the type of disease and the mode of zinc supplementation itself. Although the increase in DCX+ cells in the hippocampus of BTBR mice after the zinc supplementation in our results was not statistically significant, a trend toward increased DCX+ cells was observed. Simultaneously, changes in hippocampal neurogenesis are also associated with hippocampal excitability, as evidenced by reports that increased adult hippocampal neurogenesis reduces photostimulation-evoked hippocampal excitability [33]. Contrastingly, hippocampal excitability is increased in mice with adult hippocampal neurogenesis [33]. This may explain the decreased convulsive latency in BTBR mice, while zinc water significantly increased convulsive latency in BTBR mice.

This study has some limitations. First, the molecular mechanisms by which zinc modulates neurogenesis and the potential relationship between excitability and neurogenesis warrant further investigation. Second, this study did not detect change in hippocampal zinc concentrations after zinc supplementation. Additionally, a male-to-female ratio of approximately 4.5:1 in children with ASD was reported in clinical studies [29]. This difference was also found in animal models of ASD, where the impairment of social competence in women is generally less pronounced than in men [68, 69]. To avoid sex differences, only male mice were selected in this study, and it is necessary to further compare the effects of sex on the behavioral phenotypes of ASD. It should also be noted that long-term high-concentration zinc supplementation damages the health of laboratory mice. Moreover, zinc concentration was closely related to changes in the behavior of the experimental mice. It is necessary to further study the relationship between dose, time of administration initiation, duration of administration, and ameliorative effect. These questions should be explored in future research.

In conclusion, this study suggests that the remission of autism-like behavior in BTBR mice by zinc water may be related to the proliferation of hippocampal neural precursor cells and the regulation of excitability, providing new evidence for the prevention of ASD with zinc supplementation.

## Supplementary Information

Supplementary material (DOCX 21 kb)
