# Supplementary material for: Zinc Water Prevents Autism-Like Behaviors in the BTBR Mice
Source: Biol Trace Elem Res. 2023 Jan 5;201(10):4779–92. doi: 10.1007/s12011-022-03548-1 (PMC10415509; doi:10.1007/s12011-022-03548-1)
Supplement: Supplementary file 1 — (DOCX 21 kb) [file 12011_2022_3548_MOESM1_ESM.docx]

Supplementary Material

**Animals**

Behavior group n=12, each mouse tested 5 experimental contents (Open field test, Self-grooming, Marble-burying, Y-maze, Three-chamber experiment). After the test was completed, the samples were collected and stored at -80°C for later use. In addition, n=12 was only used for convulsion threshold detection. n=4-6 for immunofluorescence only. n=4 for Timm staining only.

Table 1 **Content of metallic elements in feed**

| Metal elements | Content |
| --- | --- |
| Zinc | 30mg/kg |
| Iron | 37mg/kg |
| Calcium | 5.0g/kg |
| Copper | 6.0mg/kg |

**Measurement of zinc concentration in serum**

1. Take 10mL of Reagent II and add it to a bottle of Reagent III before use, and dissolve it by shaking for at least 30min. After dissolving, it can be stored at 4℃ for a week.

2. Preheat the spectrophotometer for 30min and adjust the wavelength to 620nm.

3. Follow the table below.

Table 2 Zinc concentration in serum

| Name(μL) | Blank tube | Standard tube | Measurement tube |
| --- | --- | --- | --- |
| Distilled water | 50 | - | - |
| Standard solution | - | 50 | - |
| Serum | - | - | 50 |
| Reagent I | 100 | 100 | 100 |
| Mix; 10000rpm×10min, room temperature | | | |
| Supernatant | 100 | 100 | 100 |
| Reagent III | 200 | 200 | 200 |

Mix thoroughly and let stand at room temperature for 10 min. Aspirate 200μL of supernatant in a 96-well plate, measure the absorbance at 620 nm, and record as A _Blank_, A _Standard,_ and A _Measurement_.

4．Calculation of zinc concentration in serum.

Zinc concentration in serum(mmol/L) =C _Standard_ × (A _Measurement_-A _Blank_)/ (A _Standard_-A _Blank_)

C _Standard_ =0.2 mmol/L

**Table 3 Modified Racine Scale**[1]

| Description of a convulsive seizure | Levels |
| --- | --- |
| No convulsions | 0 |
| Closed eyes, whisker movement, mouth, nose and facial muscle clonus | 1 |
| Rhythmic head nodding on top of level 1. | 2 |
| Forelimb clonus on the basis of level 2. | 3 |
| Hind limb standing on the basis of level 3. | 4 |
| Fall seizure on the basis of level 4 | 5 |

**Table 4 Gavazos MFS Standards**[2]

| Staining characteristics | Scores |
| --- | --- |
| No granules | 0 |
| Occasional granules | 1 |
| Occasional to moderate granules | 2 |
| Prominent granules | 3 |
| Continuous distribution of protruding particles, or highly concentrated bands of particles with continuous or near-continuous distribution. | 4 |
| Continuous or nearly continuous bands of densely layered particles | 5 |

**Reference**

1. Cavazos JE, Golarai G, Sutula TP. **Mossy fiber synaptic reorganization induced by kindling: time course of development, progression, and permanence.** J Neurosci. 1991 Sep;11(9):2795-803. *https://* *doi: 10.1523/JNEUROSCI.11-09-02795.1991.*

2. Luttjohann A, Fabene PF, van Luijtelaar G: **A revised Racine's scale for PTZ-induced seizures in rats**. *Physiol Behav* 2009, **98**(5):579-586 *https://doi.org/10.1016/j.physbeh.2009.09.005*.
